# Early B-cell factor 3 (*EBF3*) is a novel tumor suppressor gene with promoter hypermethylation in pediatric acute myeloid leukemia

**DOI:** 10.1186/s13046-014-0118-1

**Published:** 2015-01-22

**Authors:** Yan-Fang Tao, Li-Xiao Xu, Jun Lu, Shao-Yan Hu, Fang Fang, Lan Cao, Pei-Fang Xiao, Xiao-Juan Du, Li-Chao Sun, Zhi-Heng Li, Na-Na Wang, Guang-Hao Su, Yan-Hong Li, Gang Li, He Zhao, Yi-Ping Li, Yun-Yun Xu, Hui-Ting Zhou, Yi Wu, Mei-Fang Jin, Lin Liu, Xue-Ming Zhu, Jian Ni, Jian Wang, Feng Xing, Wen-Li Zhao, Jian Pan

**Affiliations:** Department of Hematology and Oncology, Children’s Hospital of Soochow University, Suzhou, China; Department of Gastroenterology, the 5th Hospital of Chinese PLA, Yin chuan, China; Department of Cell and Molecular Biology, Cancer Institute (Hospital), Chinese Academy of Medical Sciences, Peking Union Medical College, Beijing, China; Translational Research Center, Second Hospital, The Second Clinical School, Nanjing Medical University, Nanjing, China

**Keywords:** Early B-cell factor 3, Pediatric acute myeloid leukemia, Methylation, Tumor suppressor, Real-time PCR array

## Abstract

**Background:**

Pediatric acute myeloid leukemia (AML) comprises up to 20% of all childhood leukemia. Recent research shows that aberrant DNA methylation patterning may play a role in leukemogenesis. The epigenetic silencing of the *EBF3* locus is very frequent in glioblastoma. However, the expression profiles and molecular function of *EBF3* in pediatric AML is still unclear.

**Methods:**

Twelve human acute leukemia cell lines, 105 pediatric AML samples and 30 normal bone marrow/idiopathic thrombocytopenic purpura (NBM/ITP) control samples were analyzed. Transcriptional level of *EBF3* was evaluated by semi-quantitative and real-time PCR. *EBF3* methylation status was determined by methylation specific PCR (MSP) and bisulfite genomic sequencing (BGS). The molecular mechanism of *EBF3* was investigated by apoptosis assays and PCR array analysis.

**Results:**

*EBF3* promoter was hypermethylated in 10/12 leukemia cell lines. Aberrant *EBF3* methylation was observed in 42.9% (45/105) of the pediatric AML samples using MSP analysis, and the BGS results confirmed promoter methylation. *EBF3* expression was decreased in the AML samples compared with control. Methylated samples revealed similar survival outcomes by Kaplan-Meier survival analysis. *EBF3* overexpression significantly inhibited cell proliferation and increased apoptosis. Real-time PCR array analysis revealed 93 dysregulated genes possibly implicated in the apoptosis of *EBF3*-induced AML cells.

**Conclusion:**

In this study, we firstly identified epigenetic inactivation of *EBF3* in both AML cell lines and pediatric AML samples for the first time. Our findings also showed for the first time that transcriptional overexpression of *EBF3* could inhibit proliferation and induce apoptosis in AML cells. We identified 93 dysregulated apoptosis-related genes in *EBF3*-overexpressing, including DCC, AIFM2 and DAPK1. Most of these genes have never been related with *EBF3* over expression. These results may provide new insights into the molecular mechanism of *EBF3*-induced apoptosis; however, further research will be required to determine the underlying details.

Our findings suggest that *EBF3* may act as a putative tumor suppressor gene in pediatric AML.

## Background

Acute myeloid leukemia (AML) is a type of cancer that arises from the myeloid cell. It is the most common form of acute leukemia in adults and the second most common form of leukemia in children after acute lymphoblastic leukemia (ALL) [1,2]. Pediatric AML comprises up to 20% of all childhood leukemia and the mechanism behind poor survival of acute myeloid leukemia (AML) patients remains unclear [3]. Several novel recurrent mutations have been found to involve epigenetically regulated genes in AML, including *DNMT3A* [4,5], *TET2* [6,7], and *IDH1/2* [8], which are involved in the regulation of DNA methylation, and *EZH2* [9,10] and *ASXL-1* [11], which are implicated in the regulation of histones [11]. Importantly, the presence of *DNMT3A*, *IDH1*, or *IDH2* mutations may confer sensitivity to novel therapeutic approaches, including the use of demethylating agents. We propose that understanding the role of methylation in AML will lead to more rational therapeutic approaches targeting this disease [4,12].

One important role of epigenetic regulation is that it affects gene expression; recent research has shown that aberrant DNA methylation may play a role in leukemogenesis [13]. DNA methylation is an important regulator of gene transcription. DNA methylation is an epigenetic modification that typically occurs at CpG (cytosine-phosphate-guanine) sites in mammalian cells [14]. The prognostic impact of global DNA methylation and hydroxymethylation has been assessed and global DNA methylation predicted overall survival in myelodysplastic syndromes [15]. The importance of epigenetic aberrations in the pathogenesis of leukemias has been revealed by recurrent gene mutations that highlight epigenetic pathways as well as by the clinical success of therapies like 5-azacytidine and decitabine that work through epigenetic mechanisms. Azacitidine seems effective in WHO-AML, including patients with >30% BM blasts [16]. Multiple clinical trials have shown the promising activity of low-dose decitabine in AML, MDS, CML, and hemoglobinopathies, whereas its efficacy in solid tumors is rather limited. Recent clinical trials have investigated new dosing schedules, routes of administration, and combination of decitabine with other agents, including histone deacetylase (HDAC) inhibitors [17].

The early B-cell factors (EBF) are a family of four highly conserved DNA-binding transcription factors with an atypical zinc-finger and helix-loop-helix motif. EBF proteins have diverse functions in the development of multiple lineages, including neurons, B cells, and adipocytes. B lymphocytes are generated from hematopoietic stem cells in a series of steps controlled by transcription factors. One of the most important regulators of this process is early B cell factor (EBF). EBF and closely related proteins (EBF2, *EBF3*, EBF4, Collier/Knot and Unc-3) constitute a novel transcription factor family (here, termed the EBF family). All members of the EBF family possess a highly conserved DNA-binding domain (DBD) that is distinct from that of other known DNA-binding proteins. Multiple lines of evidence indicate that expression of EBF is a principle determinant of the B cell fate [18,19]. EBF activity is important for both stabilizing commitment and driving aspects of differentiation in Xenopus muscle cells [20]. Alterations in various developmental pathways are common themes in cancer. Accumulating evidence indicates that genomic deletion of the *EBF1* gene contributes to the pathogenesis, drug resistance, and relapse of B-progenitor acute lymphoblastic leukemia (ALL) [21-23]. Epigenetic silencing and genomic deletion of the *EBF3* locus on chromosome 10q are very frequent in glioblastoma (GBM). Strikingly, the frequency of *EBF3* loss in GBM is similar to the loss of *PTEN*, a key suppressor of gliomagenesis. Cancer-specific somatic mutations were detected of *EBF3* in GBM and both *EBF1* and *EBF3* in pancreatic ductal adenocarcinoma [24]. In a genome-wide screen for putative tumor suppressor genes, the *EBF3* locus on the human chromosome 10q26.3 was found to be deleted or methylated in 73% of brain tumor cases. Silencing of the *EBF3* locus has been observed in brain, colorectal, breast, liver, and bone tumor cell lines, and its reactivation was achieved with 5-aza-2′-deoxycytidine and trichostatin A treatment in a significant portion of these tumor cells [25]. In gastric carcinoma, inactivation of the *EBF3* gene is frequently accompanied by promoter hypermethylation in several gastric cancer cell lines. Promoter methylation of *EBF3* was detected in 42/104 (40.4%) gastric cancer tissues but not in normal gastric tissues. These results suggest that the *EBF3* tumor suppressor is epigenetically silenced and that it serves as an independent prognostic marker in gastric carcinoma [26]. Therefore, *EBF3* regulates a transcriptional program underlying a putative tumor suppression pathway [25]. Likewise, the expression of *EBF3* results in cell cycle arrest and apoptosis. A previous study has shown that the expression of cyclin-dependent kinase inhibitors was profoundly affected upon early activation and then repression of p21 (cip1/waf1) and persistent activation of both p27 (kip1) and p57 (kip2), whereas genes involved in cell survival and proliferation were suppressed [25].

However, reports on the methylation status of *EBF3* in the blood system are rare, and its expression and role in pediatric AML remains unclear. The aim of this study was to analyze the methylation profile and molecular function of *EBF3* in pediatric AML. Identifying aberrant methylated genes may provide better understanding of the pathogenesis of AML [27], thereby paving the way for the development of novel tumor markers and therapeutic targets.

## Methods

### Cell lines

Leukemia cell lines HL-60, MV4-11, U937, THP-1 and K562 were obtained from the American Type Culture Collection (ATCC). CCRF, Raji, Jurkat, 697, Daudi and SHI-1 cell lines (gifts from Professor Wang Jian-Rong, The Cyrus Tang Hematology center of Soochow University) [28], NALM-6 cell lines (gifts from Professor Tang Yong-Ming, Zhejiang University). HL-60, MV4-11, U937, THP-1 and SHI-1 are AML cell lines. All cell lines were maintained at 37°C in the RPMI 1640 (GibcoR, Life Technologies, Carlsbad, CA) supplemented with 10% fetal bovine serum (Invitrogen, Life Technologies, Carlsbad, CA).

### Patients and samples

Bone marrow specimens were obtained at the time of diagnosis during routine clinical assessment of 105 pediatric patients with AML, who presented at the Department of Hematology and Oncology, Children’s Hospital of Soochow University between 2000 and 2011. Ethical approval was provided by the Children’s Hospital of Soochow University Ethics Committee (No.SUEC2000-021 & No.SUEC2011-037), and written informed consent was obtained from the parents or guardians. AML diagnosis was made in accordance with the revised French–American–British (FAB) classification. The main clinical and laboratory features of the patient cohort are summarized in Table 1. Additionally, bone marrow samples from 23 healthy donors and 7 patients with Idiopathic thrombocytopenic purpura (ITP) were analyzed as controls. Bone marrow mononuclear cells (BMNCs) were isolated using Ficoll solution within 2 h after bone marrow samples harvested and subjected for the extraction of total RNA and genomic DNA.

**Table 1:** **Association of** ***EBF3*** **expression with clinico-pathological characteristics in 105 pediatric AML samples**

**Clinical variables**	**No. of patients**	***EBF3*** **expression (n)**		**P**
		**Low**	**High**	
Gender				
Male	42	19	23	0.473
Female	63	33	30	
Age (years)				
<6	60	29	31	0.778
≥6	45	23	22	
Leukocyte (/μl)				
>10000	61	32	29	0.479
≤10000	44	20	24	
FAB				
M1-M6	93	47	46	0.563
M7	12	5	7	
Cytogenetics				
Favorable	50	23	27	0.502
Intermediate	27	16	11	
Unfavorable	28	13	15	
MRD				
<0.25%	49	28	21	0.114
≥0.25%	56	24	32	

### CD34^+^ cell purification

For CD34 ^+^cell selection, the Miltenyi immunoaffinity device (VarioMACS 130-046-703) was used according to the manufacturer’s instructions (Miltenyi Biotech, Auburn, CA). Briefly, the CD34^+^ cells are magnetically labeled with CD34 MicroBeads. Then, the cell suspension is loaded onto a MACSR Column which is placed in the magnetic field of a MACS Separator. The magnetically labeled CD34^+^ cells are retained within the column. The unlabeled cells run through; CD34^+^ cells were adsorbed on the magnetic poles. After removing the column from the magnetic field, the magnetically retained CD34^+^ cells can be eluted as the positively selected cell fraction.

### Sodium bisulfite modification of genomic DNA

High-molecular-weight genomic DNA was extracted from cell lines and biopsies by a conventional phenol/chloroform method. The sodium bisulphite modification procedure was as described [29] with slight modification. In brief, 600 ng of genomic DNA was denatured in 3 M NaOH for 15 min at 37°C, then mixed with 2 volumes of 2% low-melting-point agarose. Agarose/DNA mixtures were then pipetted into chilled mineral oil to form agarose beads. Aliquots of 200 μl of 5 M bisulphite solution (2.5 M sodium metabisulphite, 100 mM hydroquinone, both Sigma, USA) were added into each tube containing a single bead. The bisulphite reaction was then carried out by incubating the reaction mixture for 4 h at 50°C in the dark. Treatments were stopped by equilibration against 1 ml of TE buffer, followed by desulphonation in 500 μl of 0.2 M NaOH. Finally, the beads were washed with 1 ml of TE buffer and directly used for PCR.

### Methylation-specific PCR

The methylation status of the *EBF3* promoter region was determined by methylation-specific PCR. Primers distinguishing unmethylated (U) and methylated (M) alleles were designed to amplify the sequence:

- *EBF3* M-forward: 5- TAGGAATTTTGTTATGTGTGAGGTC-3;
- *EBF3* M-reverse: 5- AAATACCGTTATTAATTTTCTCGTT-3;
- *EBF3* U-forward: 5- TAGGAATTTTGTTATGTGTGAGGTT-3;
- *EBF3* U-reverse: 5- AATAAATACCATTATTAATTTTCTCATT-3.

Each PCR reaction contained 20 ng of sodium bisulphite-modified DNA, 250 pmol of each primer, 250 pmol deoxynucleoside triphosphate, 1 × PCR buffer, and one unit of ExTaq HS polymerase (Takara, Tokyo, Japan) in a final reaction volume of 20 μl. Cycling conditions were initial denaturation at 95°C for 3 min, 40 cycles of 94°C for 30 s, 63°C (M) or 58°C (U) for 30 s, and 72°C for 30 s. For each set of methylation-specific PCR reactions, in vitro-methylated genomic DNA treated with sodium bisulphite served as a positive methylation control. PCR products were separated on 4% agarose gels, stained with ethidium bromide and visualized under UV illumination. For cases with borderline results, PCR analyses were repeated.

### Bisulfite genomic sequencing

Bisulfite genomic sequencing (BGS) was performed as previously described [30]. BGS primers were from +709 to +1031 including 20 CpGs. *EBF3* F: 5- TTAGGAATTTTGTTATGTGTGAGGT −3 and *EBF3* R: 5- TTATATTTTATTTTCCTTCTATACCATAAA −3. Amplified BGS products were TA-cloned; and five to six randomly chosen colonies were sequenced. DNA sequences were analyzed with QUMA Analyzer. (http://quma.cdb.riken.jp/).

### Leukemia cells treated with 5-aza-2′-deoxycytidine

De-methylation was induced with 5-aza-dC (5-Aza, Sigma-Aldrich, St Louis, MO, USA) treatment at a concentration that induced de-methylation of the DNA without killing the cells. Culture media for HL-60 and MV4-11 cells contained 5 μM or 10uM 5-Aza. DNA and RNA were extracted after 72 hours of 5-Aza treatment for the following analysis.

### Quantitative reverse-transcription PCR for *EBF3*

Quantitative real-time PCR was performed to determine the expression levels of *EBF3* genes. Total RNA was reverse transcribed using the Reverse Transcription Kit, according to the manufacturer’s protocol (Applied Biosystems Inc., Foster City, CA). The real time PCR primers used to quantify GAPDH expression were: F: 5′-AGAAGGCTGGGGCTCATTTG-3′ and R: 5′-AGGGGCCATCCACAGTCTTC-3′ and for *EBF3* were: F: 5′- ATGGCTCCTCCGCTAACTCT-3′ and R: 5′- TCCGTCCTTTGATGCTGGGT-3′. Expression of *EBF3* was normalized to endogenous GAPDH expression.

### *EBF3* lentiviral expression constructs and lentivirus production

Briefly, an approximately 1650 bp fragment containing the human *EBF3* gene was directly cloned into the pMD18-T vector. Positive clones were confirmed by sequencing and subcloned into the pLVX-IRES-ZsGreen vector (Clontech Laboratories, Inc. Tokyo, Japan). The vector plasmids, pLVX-IRES-ZsGreen1, pLP1, pLP2 and pLP/VSVG were amplified in E.Coli and purified using the Endofree Maxiprep Kit (QIAGEN, Inc. Duesseldorf, German). 270 μg of transfer vector, 176 μg of pLP1, 95 μg of pLP/VSVG and 68 μg of pLP2 was mixed with 0.25 M CaCl2 (Sigma-Aldrich, St Louis, MO, USA) and added to same volume of 2 × HEPES (Sigma-Aldrich, St Louis, MO, USA) and mixed while bubbling for 20 min to allow a precipitate to form. This was then added to a 175 cm2 flask of approximately 60% confluent 293 T cells containing 20 mL DMEM supplemented with 10% fetal calf serum, 100 U/mL penicillin, 100 μg/mL streptomycin and 2 mM glutamine and incubated for 48 h at 37°C in 5% CO_2_. The supernatant was centrifuged at 1,700 g for 10 min to pellet cell debris, and ultracentrifuged at 121,603 g for 2 h. The pellet containing concentrated virus was resuspended in DMEM without supplements and stored at −80°C.

### Cell proliferation analysis

Acute myeloid leukemia cells were seeded in 96-well plates at 2 × 10^4^ cells per well. 20 μl CCK-8(Dojindo Molecular Technologies, Tokyo, Japan) was added to each well and incubated at 37°C for a further 4 hours. The optical density (OD) values were measured at 450 nm on a scanning multi-well spectrophotometer (BioRad Model 550, USA). Compared with the control group, cell proliferation was calculated as proliferation values. All experiments were performed in triplicate and repeated twice. The results were analyzed using ANOVA and the Student-Newman-Keuls tests, *p* < 0.05 were considered significant.

### Apoptosis assay

Apoptosis assay was according to the manual operation of BD Annexin V Staining Kit (Cat: 556420, BD Biosciences, Franklin Lakes and NJ USA). Briefly, wash cells twice with cold PBS and then resuspend cells in 1 × Binding Buffer at a concentration of ~1 × 10^6^ cells/ml. Transfer 100 μl of the solution (~1 × 10^5^ cells) to a 5 ml culture tube. Add Annexin V and PI 5 μl/test. Gently mix the cells and incubate for 15 min at RT in the dark. Add 400 μl of 1 × Binding Buffer to each tube. Analyzed by flow cytometry as soon as possible (within 1 hour).

### Western blot analysis

For western blot analysis, cellular proteins were extracted in 40 mM Tris–HCl (pH 7.4) containing 150 mM NaCl and 1% (v/v) Triton X-100, supplemented with a cocktail of protease inhibitors. Equal amounts of protein were resolved on 12% SDS-PAGE gels, and then transferred to a PVDF membrane (Millipore, Bedford, MA). Blots were blocked and then probed with antibodies against PARP (1:1000,9532 s, Cell Signaling Technology, Inc. Danvers, MA), Caspase3 (1:1000,9665 s, Cell Signaling Technology, Inc.Danvers, MA), Caspase9 (1:1000,9505 s, Cell Signaling Technology, Inc. Danvers, MA), *EBF3* (1:1000, ab122917, Abcam, Cambridge, MA Office, USA), GAPDH (1:5000, G8795, Sigma, St. Louis, MO). AIFM2 (1:1000, sc-377120, Santa Cruz Biotechnology, Inc. Dallas, Texas, USA), BIRC8 (1:1000, sc-130107, Santa Cruz Biotechnology, Inc. Dallas, Texas, USA), BCL2L11 (1:1000, sc-8267, Santa Cruz Biotechnology, Inc. Dallas, Texas, USA), CDKN1A (1:1000, 2947 s, Cell Signaling Technology, Inc. Danvers, MA). After washing, the blots were incubated with horseradish peroxidase-conjugated secondary antibodies and visualized by enhanced chemiluminescence kit (Pierce, Rockford, IL). Protein bands were visualized after exposure of the membrane to Kodak X-ray film.

### Real-time PCR array analysis

For RNA extraction, cells were immediately submerged in 2 ml Trizol (Invitrogen Co., NY, USA), stored at −80°C until further processed. A volume of 1 ml of each sample was spun at 4°C for 15 min at 12,000 g to remove debris and DNA, 1 ml of supernatant was mixed with 200 ul chloroform, shaken for 15 seconds, incubated at Room Temperature for 2–3 minutes and spun for 10 minutes at 12,000 g at 4°C. RNA was precipitated by adding 500 μl of the aqueous phase to an equal volume of isopropanol and spun at 14,000 g at 4°C for 10 minutes. RNA was washed with 75% ethanol, spun at 14,000 g at 4°C for 10 minutes, dried and resuspended in 40 μl DEPC-treated H_2_O. The final RNA concentration was determined using a spectrophotometer (Nanodrop 2000) and the purity was assessed by agarose gel electrophoresis. cDNA synthesis was performed on 4 μg of RNA in a 10 μl sample volume using SuperScript II reverse transcriptase (Invitrogen Co., NY, USA) as recommended by the manufacturer. The RNA was incubated with 0.5 μg of oligo(dT)12–18mers primers (Invitrogen Co., NY, USA) for 7 minutes at 70°C and then transferred onto ice. Then, 9 μl of a master mix containing 4 μl of SuperScript II buffer, 2 μl of 0.1 M DTT, and 1 μl each of dNTPs stock (10 mM), Rnasin (40 UI) and SuperScript II were added to the RNA sample, spun and incubated at 42°C for 60 min followed by 5 min at 70°C to inactivate the enzyme. cDNA was stored at −20°C. Real-time PCR array (SABioscience Human Apoptosis PCR Array PAHS-3012) analysis was performed in a total volume of 20 μl including 2 μl of cDNA, primers (0.2 mM each) and 10 μl of SYBR Green mix (Roche Co., Basel, Switzerland.). Reactions were run on an Light cycler 480 using the universal thermal cycling parameters (95°C 5 min, 45 cycles of 10 sec at 95°C,20 sec at 60°C and 15 sec at 72°C; melting curve: 10 sec at 95°C, 60 sec at 60°C and continues melting). Results were obtained using the sequence detection software Light cycler 480 and analyzed using Microsoft Excel. For all samples melting curves were acquired for quality control purposes. For gene expression quantification, we used the comparative Ct method. First, gene expression levels for each sample were normalized to the expression level of the housekeeping gene encoding Glyceraldehydes 3-phosphate dehydrogenase (GAPDH) within a given sample (−⊿Ct); the relative expression of each gene was calculated with10^6^ *Log2(−⊿Ct ). The difference between the *EBF3* over-expression samples compared to the control samples was used to determine the10^6^ *Log2(−⊿Ct ). Statistical significance of the gene expression difference between the *EBF3* over-expression and the control samples was calculated with the T-test using SPSS 11.5 software.

### Ingenuity pathway analysis (IPA)

Datasets representing genes with altered expression profile derived from Real-time PCR array analyses were imported into the Ingenuity Pathway Analysis Tool (IPA Tool; Ingenuity H Systems, Redwood City, CA, USA; http://www.ingenuity.com). In IPA, differentially expressed genes are mapped to genetic networks available in the Ingenuity database and then ranked by score. The basis of the IPA program consists of the Ingenuity Pathway Knowledge Base (IPKB) which is derived from known functions and interactions of genes published in the literature. Thus, the IPA Tool allows the identification of biological networks, global functions and functional pathways of a particular dataset. The program also gives the significance value of the genes, the other genes with which it interacts, and how the products of the genes directly or indirectly act on each other, including those not involved in the microarray analysis. The networks created are ranked depending on the number of significantly expressed genes they contain and also list diseases that were most significant. A network is a graphical representation of the molecular relationships between molecules. Molecules are represented as nodes, and the biological relationship between two nodes is represented as an edge (line). All edges are supported by at least 1 reference from the literature, from a textbook, or from canonical information stored in the Ingenuity Pathways Knowledge Base.

### Statistical analysis

SPSS v11.5 (SPSS Inc., Chicago, IL) was used for statistical analysis. Data are presented as means ± standard deviation. Group t-test was used to compare the expression of *EBF3* between DMSO group and 5-Aza group. Statistical significance between methylated sample data and clinical pathological features of AML patients were analyzed by Pearson chi-square test or Fisher’s exact test. Statistical significance of *EBF3* expression among NBM and pediatric AML groups was determined using one-way ANOVA. A p <0.05 was considered statistically significant.

## Results and Discussion

### The ***EBF3*** promoter is hypermethylated in AML cells

**Figure 1:**
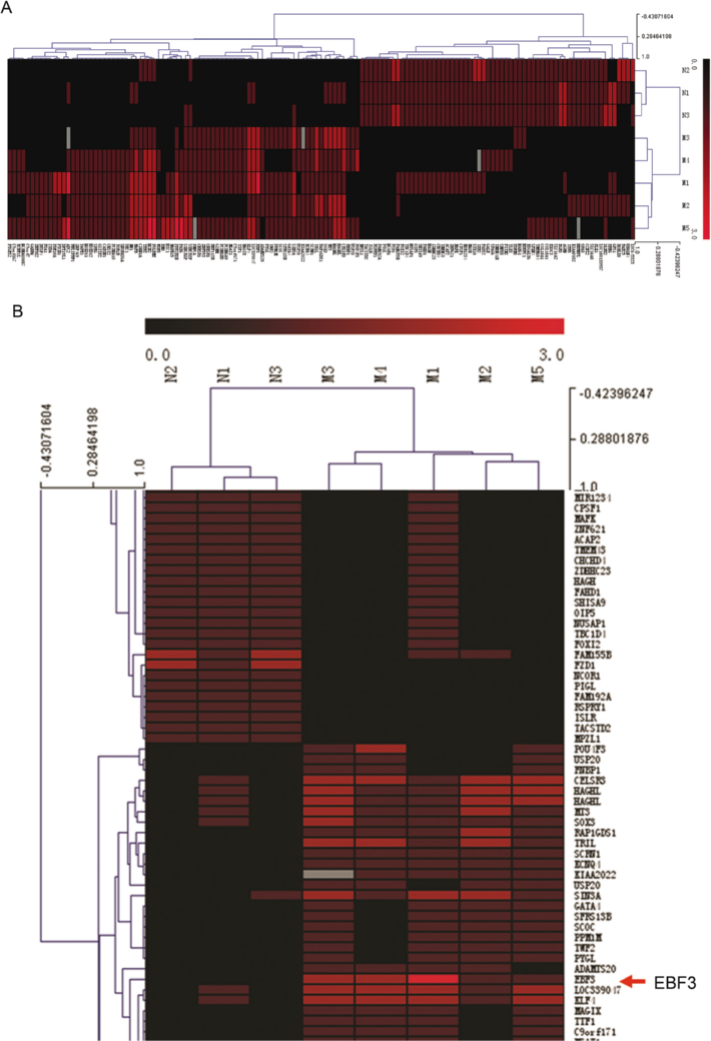
**Analysis of promoter methylation in pediatric AML by NimbleGen Human DNA Methylation arrays.** Analysis of the methylation status of genes in five pediatric AML samples (M1, M2, M3, M4 and M5) and three NBM samples (N1, N2, and N3) using NimbleGen Human DNA Methylation arrays shows that the *EBF3* promoter is significantly methylated in AML samples (5/5) and unmethylated in NBM samples (0/3). **(A)** Each red box represents the number of methylation peaks (PeakScore) overlapping the promoter region for the corresponding gene. The PeakScore is defined as the average -log10 (*P* value) from probes within the peak. **(B)** The scores reflect the probability of positive methylation enrichment.

**Figure 2:**
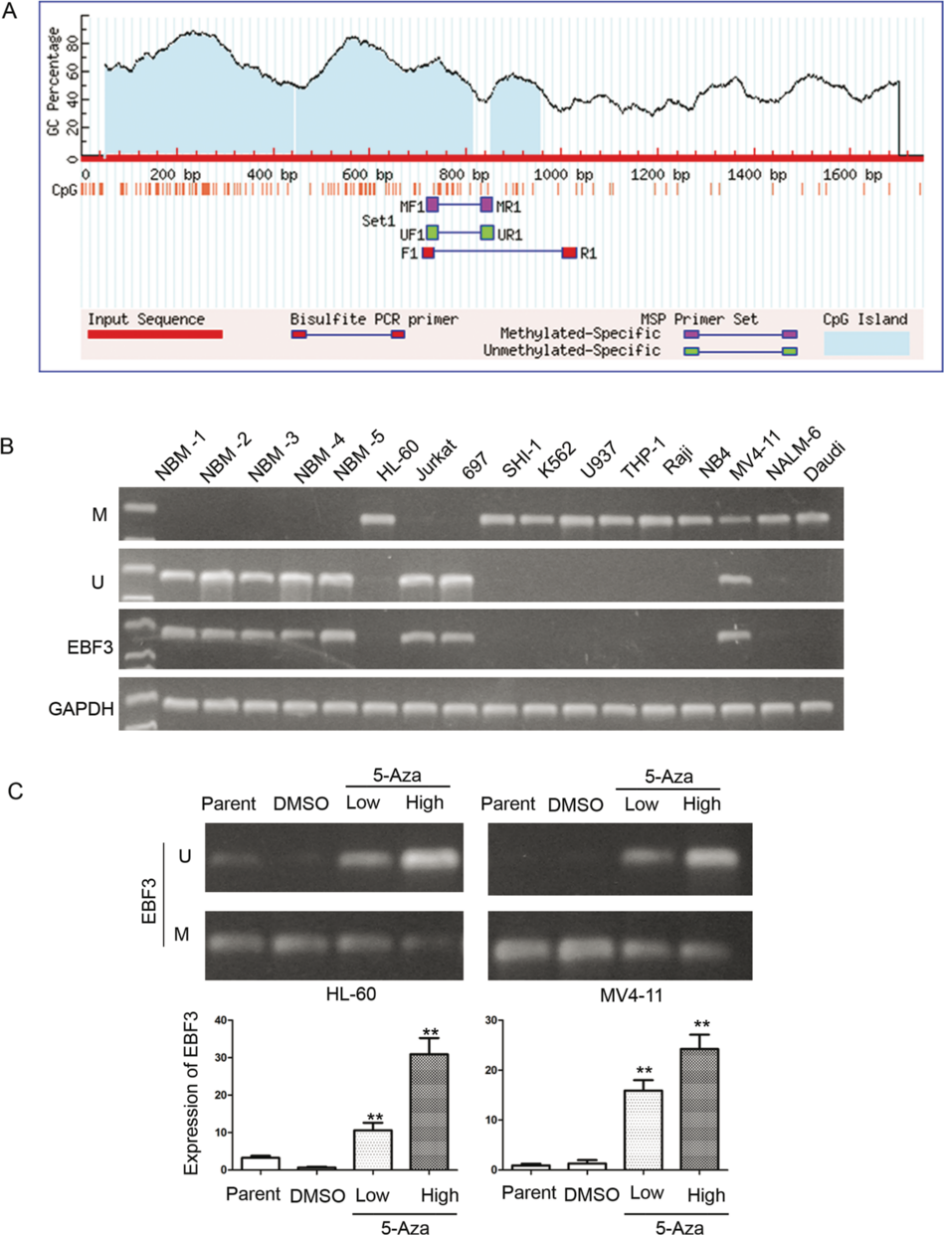
**The** ***EBF3*** **promoter is methylated in AML cell lines. (A)** Two CpG island regions can be identified in the promoter of *EBF3*. **(B)** MSP analysis of the methylation status of *EBF3* in leukemia cell lines shows that the promoter is hypermethylated in 10/12 cell lines. M and U represents MSP results using primer sets for methylated and unmethylated *EBF3* genes, respectively. **(C)** PCR analysis showed that the methylation status of *EBF3* decreased in leukemia cells following 5-Aza treatment compared with control cells treated with DMSO. The transcript level of *EBF3* is significantly upregulated in HL-60 and NB4 cells treated with 5-Aza compared with those treated with DMSO. *P < 0.05; **P < 0.01.

**Figure 3:**
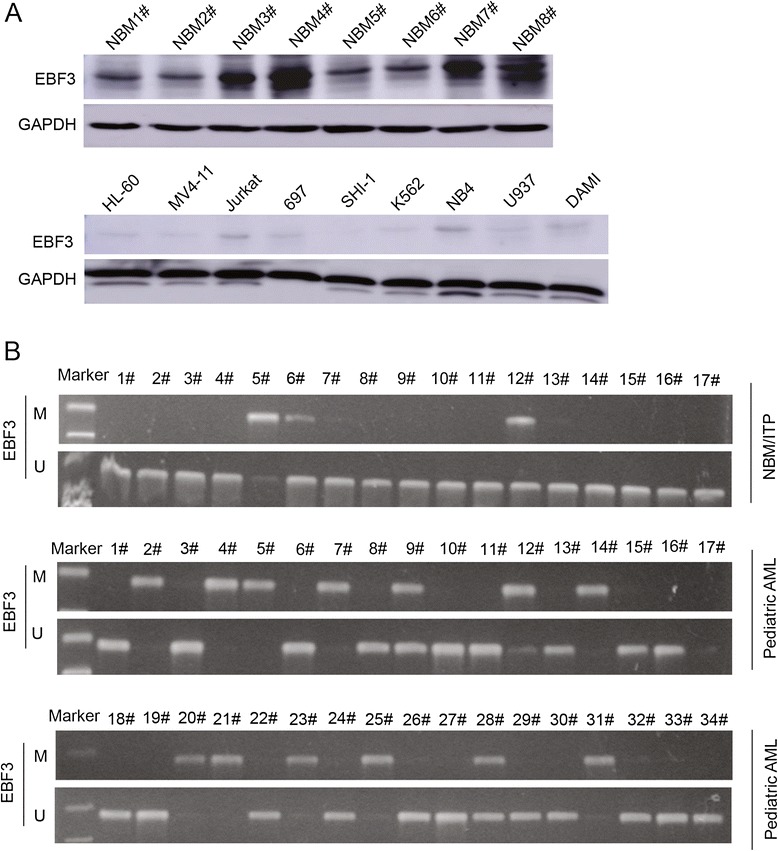
**MSP analysis showing** ***EBF3*** **promoter hypermethylation in AML samples. (A)** Western blot analysis depicting the expression of *EBF3* in eight NBM samples and nine leukemia cells. **(B)** MSP analysis the promoter methylation of *EBF3* and aberrant *EBF3* methylation was observed in 42.9% (45/105) of the pediatric AML samples compared with 13.3% (4/30) of the NBM control samples. M and U represent MSP results using primer sets for methylated and unmethylated *EBF3* genes, respectively.

Our long-term research is focused on epigenetic modifications in pediatric AML. In previous studies, we have found a series of abnormally methylated genes in AML [31,32]. We conducted CpG island array analysis to explore promoter methylation in pediatric AML. Our results implied that the *EBF3* promoter is hypermethylated in AML (Figure 1). Subsequent analysis identified two CpG islands in the *EBF3* promoter region (Figure 2A). Therefore, we conducted methylation sensitive PCR (MSP) in 12 leukemia cell lines using a primer that encompassed the CpG islands within the *EBF3* promoter. Our results showed that the *EBF3* promoter was hypermethylated in 10/12 leukemia cell lines, with the highest methylation levels observed in HL-60, NB4, SHI-1, U937(four AML cell lines) and K562 cells; whereas *EBF3* was unmethylated in 2/11 cell lines, including 697 and Jurkat cells (Figure 2B). To confirm methylation of the *EBF3* promoter, we treated the leukemia cell lines with 5-Aza. This demethylation reagent is an epigenetic modifier that inhibits DNA methyltransferase activity resulting in hypomethylation and gene activation. MSP analysis showed a decrease in *EBF3* methylation in leukemia cells following 5-Aza treatment compared with control cells treated with DMSO. In addition, we showed that *EBF3* expression was significantly upregulated in leukemia cells following 5-Aza treatment compared with control cells treated with DMSO (Figure 2C). *EBF3* expression was upregulated 50.71 fold in HL-60 cells (30.93 vs. 0.61, respectively; *P* = 0.019), and 19.00 fold in MV4-11 cells (24.27 vs. 1.28, respectively; *P* = 0.012). Western blot analysis showed significantly higher *EBF3* expression in NBM samples (n = 8) compared with leukemia cells (n = 9), which is consistent with the MSP results (Figure 3A).

### The ***EBF3*** promoter is methylated in patients with pediatric AML

**Figure 4:**
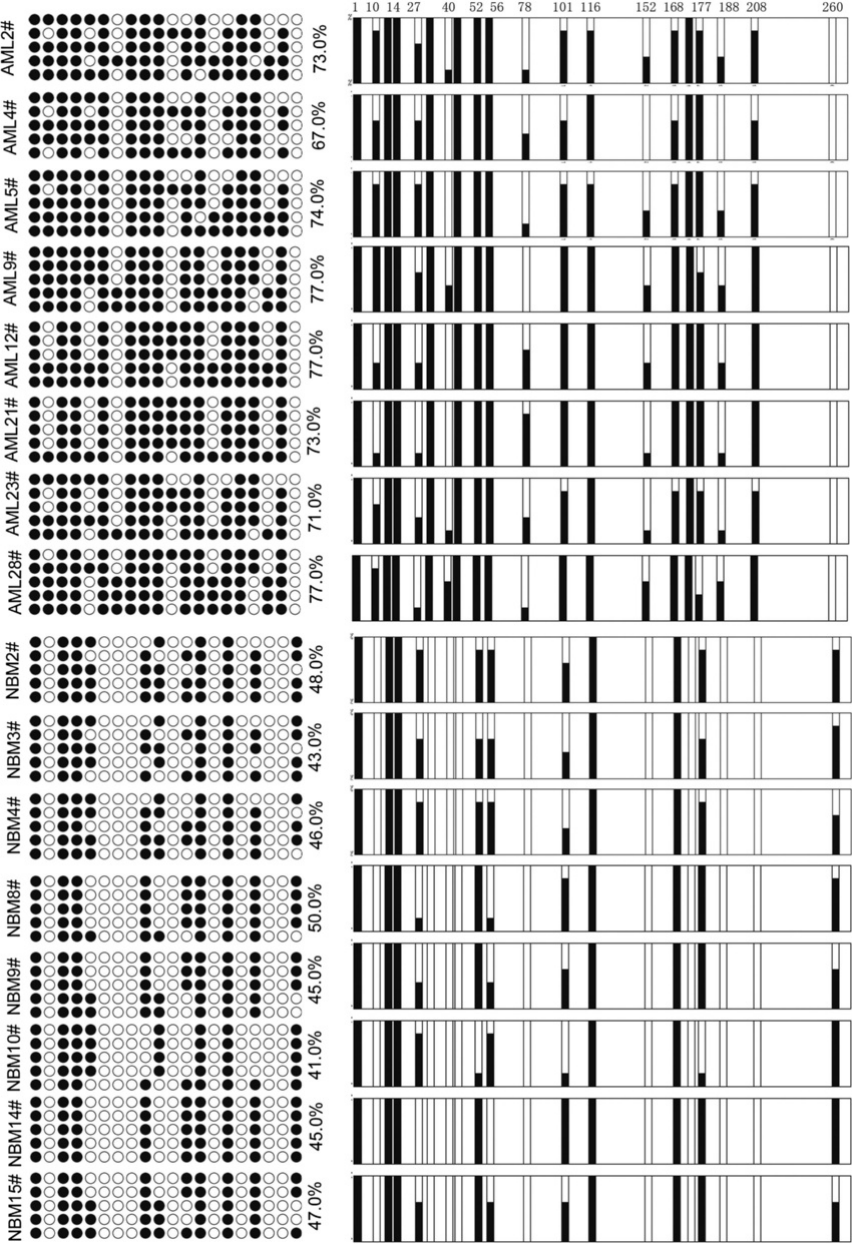
**BGS analysis depicts** ***EBF3*** **promoter hypermethylation in AML samples.** Eight NBM samples and eight AML samples were selected for further analysis by BGS. The *EBF3* promoter was methylated in the AML samples (67.0% - 77.0%); whereas the *EBF3* promoter was methylated in only 41.0% - 50.0% in the NBM samples. ● methylated cytosines; ○ unmethylated cytosines.

**Figure 5:**
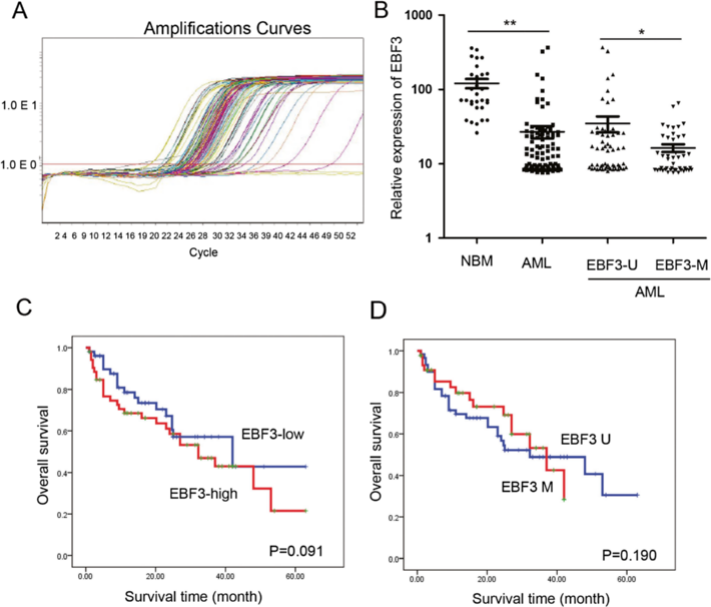
**The expression of** ***EBF3*** **was downregulated in patients with pediatric AML. (A)** Real-time PCR analysis of the transcript levels of *EBF3* in 105 pediatric AML samples and 30 NBM control samples. **(B)** Quantification shows that *EBF3* expression was found to be robustly decreased in the AML samples compared with the control samples (26.91 ± 50.86 vs. 121.14 ± 95.11, respectively; *P* <0.001). Those with methylated *EBF3* showed significantly lower levels of *EBF3* expression compared with unmethylated *EBF3* (16.32 ± 12.93 vs. 34.86 ± 65.46, respectively; *P* = 0.043). **(C)** The prognostic significance of *EBF3* expression was assessed in 105 Chinese pediatric AML patients with clinical follow-up records. Kaplan-Meier survival analysis revealed similar survival outcomes in tumors with high or low *EBF3* expression among 105 pediatric AML patients (*P* = 0.091). **(D)** Samples with *EBF3* promoter methylation revealed similar survival outcomes through Kaplan-Meier survival analysis (*P* = 0.190).

To examine the methylation status of the *EBF3* promoter in pediatric AML, we obtained samples from 105 patients with pediatric AML and 30 control patients with NBM/ITP. Aberrant *EBF3* methylation was observed in 42.9% (45/105) of the pediatric AML samples compared with 13.3% (4/30) of the NBM control samples (Figure 3B). Subsequently, eight NBM samples and eight AML samples were selected for further analysis by bisulfite genomic sequencing (BGS; Figure 4). Consistent with the MSP results, BGS confirmed that the CpG islands in the *EBF3* promoter were methylated in the AML samples (67.0% - 77.0%), whereas the CpG sites were methylated in 41.0% - 50.0% in the NBM samples. No significant differences in clinical features, such as sex, age, initial hemoglobin level, white blood cell counts, platelet counts, and chromosomal abnormalities, were observed between methylated *EBF3* samples and unmethylated samples by examination of the clinicopathologic characteristics (Table 2). Real-time qPCR was employed to examine the transcriptional levels of *EBF3* in 105 pediatric AML samples and 30 NBM/ITP control samples (Figure 5A and 5B; Table 1). *EBF3* expression was found to be robustly decreased in the AML samples compared with the control samples (26.91 ± 50.86 vs. 121.14 ± 95.11; *P* <0.001). Further analysis of the AML samples showed that 45/105 pediatric AML patients displayed *EBF3* methylation compared with 60/105 patients that exhibited unmethylated *EBF3* (Table 1). Furthermore, patients with methylated *EBF3* showed significantly lower levels of *EBF3* expression compared with patients exhibiting unmethylated *EBF3* (16.32 ± 12.93 vs. 34.86 ± 65.46; *P* = 0.043; Figure 5B). In summary, the hypermethylation status of the *EBF3* promoter in pediatric AML patient tissue was consistent with results in human myeloid leukemia cell lines. The prognostic significance of *EBF3* expression was assessed in 105 Chinese pediatric AML patients with clinical follow-up records. No significant association was found between *EBF3* expression and patient age, sex, FAB, or cytogenetics (Table 1). Kaplan-Meier survival analysis revealed similar survival outcomes in tumors with high or low *EBF3* expression among 105 pediatric AML patients (*P* = 0.091, Table 3, and Figure 5C). Multivariate analysis also suggested that expression of *EBF3* failed to be an independent prognostic factor in pediatric AML (*P* = 0.348, Table 4). The prognostic significance of *EBF3* promoter methylation was assessed by clinical follow-up records of the 105 cases of Chinese pediatric AML patients. Table 2 shows no significant differences in clinical features, such as sex, age, FAB, cytogenetics, or MRD between patients with and without methylated *EBF3* (Table 2). Samples exhibiting *EBF3* promoter methylation revealed similar survival outcomes by Kaplan-Meier survival analysis (*P* = 0.190, Table 3, and Figure 5D). Furthermore, multivariate analysis revealed that *EBF3* promoter methylation is not an independent prognostic factor in pediatric AML (*P* = 0.574, Table 4).

**Table 2:** **Association of** ***EBF3*** **promoter methylation with clinico-pathological characteristics in 105 pediatric AML samples**

**Clinical variables**	**No. of patients**	***EBF3*** **methylation (n)**		**P**
		**Negative**	**Positive**	
Gender				
Male	42	28	14	0.107
Female	63	32	31	
Age (years)				
<6	60	35	25	0.776
≥6	45	25	20	
Leukocyte (/μl)				
>10000	61	35	26	0.954
≤10000	44	25	19	
FAB				
M1-M6	93	55	38	0.250
M7	12	5	7	
Cytogenetics				
Favorable	50	27	23	0.060
Intermediate	27	12	15	
Unfavorable	28	21	7	
MRD				
<0.25%	49	28	21	1.000
≥0.25%	56	32	24

**Table 3:** **Association of** ***EBF3*** **expression/promoter methylation with Kaplan-Meier survival in 105 pediatric AML samples**

**Variable**	**No. of patients**	**Over survival**	**P**
		**Median ± SE**	
Cytogenetics			
Favorable	50	46.664 ± 3.717	<0.001
Intermediate	27	29.220 ± 3.188	
Unfavorable	28	11.161 ± 1.827	
FAB			
M1-M6	93	36.113 ± 2.885	<0.001
M7	12	8.542 ± 1.820	
Leukocyte (/μl)			
>10000	61	30.220 ± 2.974	0.803
≤10000	44	33.631 ± 4.063	
MRD			
<0.25%	49	53.627 ± 3.151	<0.001
≥0.25%	56	18.893 ± 2.425	
*EBF3* expression			
Low < 12.11	52	38.971 ± 4.402	0.091
High ≥ 12.11	53	32.864 ± 3.654	
*EBF3* methylation			
Negative	60	35.143 ± 3.509	0.190
Positive	45	29.458 ± 2.607

**Table 4:** **Cox multivariate analysis of** ***EBF3*** **expression/promoter methylation and clinico-pathological features in pediatric AML**

**Variable**	**Odds ratio**	**EXP(B) 95% CI**	**P**
Cytogenetics			
Favo vs. Inter and Unfavo	8.132	3.246(1.445-7.290)	0.004
MRD			
<0.25% vs. ≥0.25%	12.951	5.496(2.173-13.901)	0.000
Leukocyte (/μl)			
>10000 vs. ≤ 10000	1.371	1.433 (0.785-2.618)	0.242
FAB classification			
M7 vs. M1-M6	4.301	2.379(1.049-5.397)	0.038
*EBF3* Expression			
Low vs. High	0.882	0.732(0.382-1.403)	0.348
*EBF3* Methylation			
Negative vs. Positive	0.317	1.201 (0.635-2.272)	0.574

### Overexpression of *EBF3* inhibited proliferation and induced apoptosis in leukemia cells

**Figure 6:**
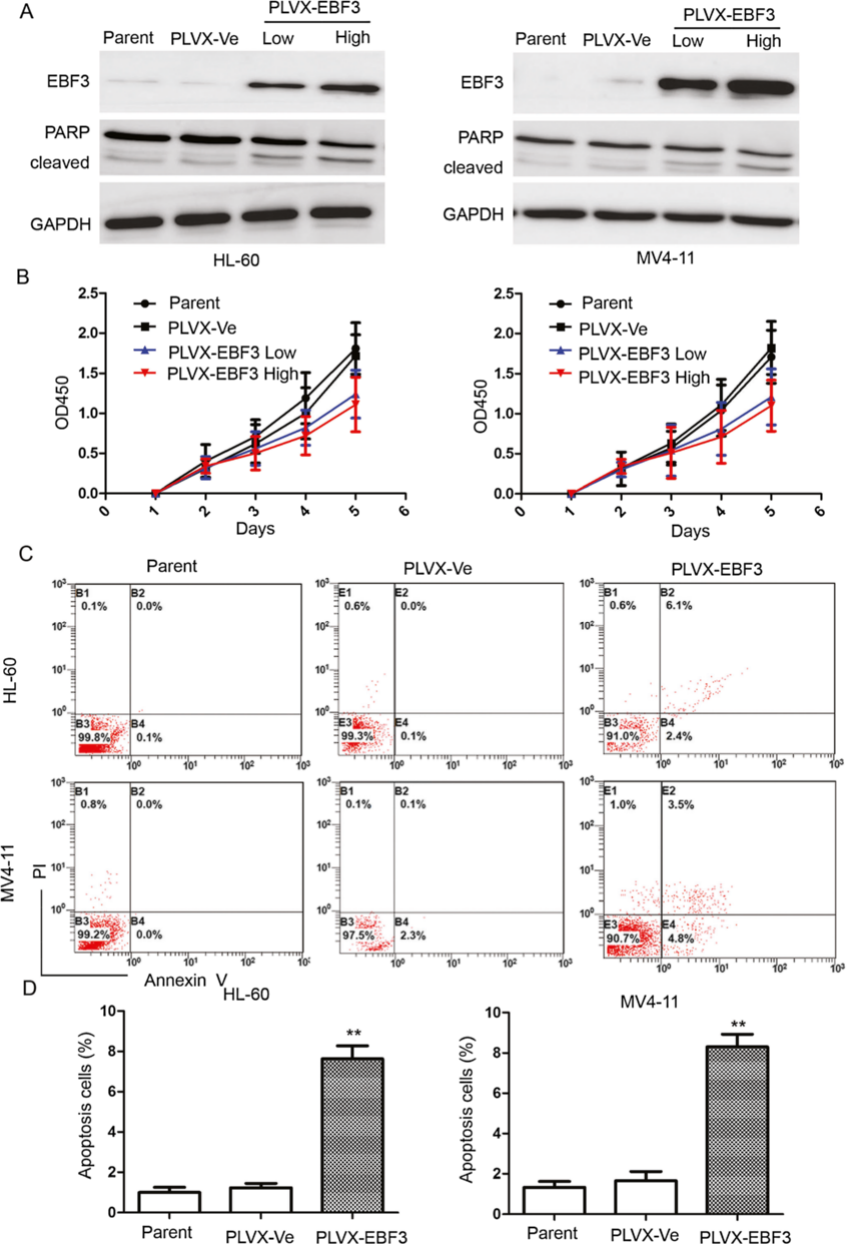
**Overexpression of** ***EBF3*** **inhibited proliferation and induced apoptosis in leukemia cells. (A)** Western blot analysis of *EBF3* expression in *EBF3* transfected leukemia cells. Transfection with *EBF3* lentivirus PLVX-*EBF3* significantly upregulated expression of *EBF3* in AML cells compared with mock-transfected cells. An expression level of cleaved PARP, a marker of apoptosis, was analyzed by Western blotting. **(B)** CCK-8 assays show that transfection with *EBF3* lentivirus inhibits proliferation in HL-60 and MV4-11 cells compared with mock-transfected cells. **(C)** The number of cells displaying apoptotic features is higher in the HL-60 and MV4-11 cells transfected with PLVX-*EBF3* compared with the mock-transfected cells. **(D)** Quantification shows that the proportion of apoptotic cells in the *EBF3*-overexpressing cells (PLVX-*EBF3*) was significantly greater than the vector control group (PLVX-Ve). **P < 0.01.

To determine whether *EBF3* is as an important player in leukemia cells, HL-60 or MV4-11 cells were stably transfected with *EBF3*. Expression of *EBF3* was significantly upregulated after transfection of the PLVX-*EBF3* lentivirus into HL-60 and MV4-11 leukemia cells (Figure 6A), and *EBF3* overexpression significantly inhibit cell proliferation (Figure 6B). A CCK-8 assay in HL-60 and MV4-11 cells showed that the inhibition rate at 5 days post-transfection was 35.4 ± 19.8% and 39.3 ± 17.6% in *EBF3*-overexpressing cells compared with the mock transfection group (*P* < 0.05). To determine whether *EBF3* induced apoptosis in leukemia cells, we performed an Annexin V assay in HL-60 and MV4-11 leukemia cells following transfection (Figure 6C and 6D). Our results showed that the proportion of apoptotic cells in the *EBF3*-overexpressing cells (PLVX-*EBF3*) was significantly greater than the vector control group (PLVX-Ve) [HL-60 (7.63% ± 1.11% vs. 1.23% ± 0.38%, respectively; *P* = 0.006) and MV4-11 (8.30% ± 1.08% vs. 1.67 ± 0.78%, respectively; *P* = 0.002]. To further confirm the apoptotic effect of *EBF3* in HL-60 and MV4-11 cells, we investigated the expression levels of cleaved PARP, a marker of apoptosis, by Western blotting. The results were consistent with the Annexin V data, confirming that *EBF3* induced apoptosis in leukemia cells (Figure 6A).

### RT-PCR array analysis showed the dysregulation of apoptosis-related genes in HL-60 cells overexpressing *EBF3*

**Figure 7:**
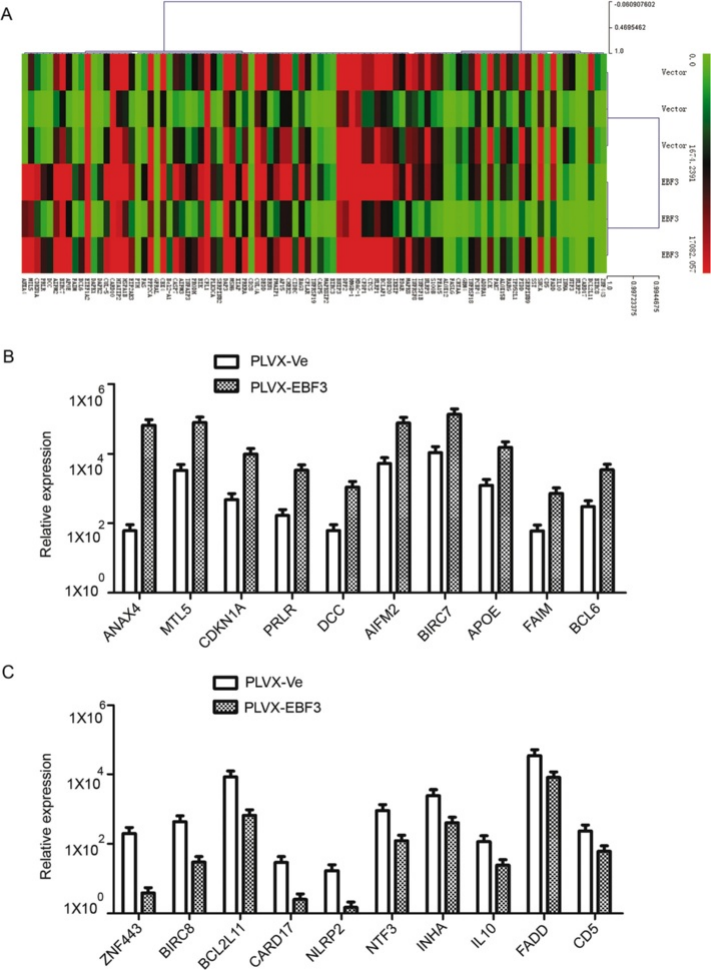
**Real-time PCR array analysis shows dysregulated genes implicated in** ***EBF3*** **over-expression. (A)** Cluster of apoptosis genes related with *EBF3* over-expression. We analyzed and clustered the expression of 370 key genes involved in apoptosis using the SABioscience Human Apoptosis PCR Array PAHS-3012 kit. **(B)** Relative expression of the genes most up-regulated in *EBF3*-overexpressing AML cells compared with mock-transfected cells. **(C)** Relative expression of the genes most down-regulated in *EBF3*-overexpressing cells compared with mock transfected cells.

**Figure 8:**
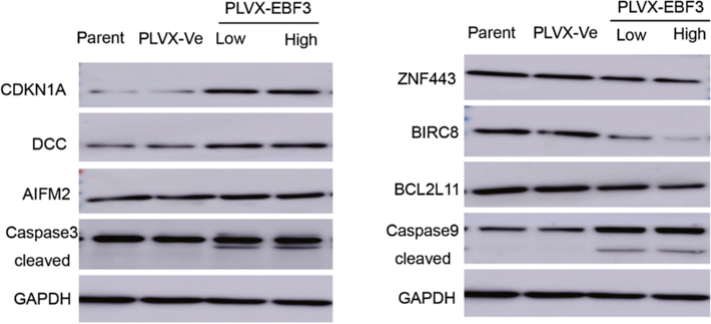
**Western-blot analysis verifying the dysregulated genes implicated in** ***EBF3*** **-overexpressing cells.** Western blot analysis of cells transfected with PLVX-*EBF3* compared with PLVX-Ve control cells. The increase of cleaved caspase-3 and caspase-9 and up-regulation of CDKN1A, DCC, and AIFM2 and the down-regulation of ZNF443, BIRC8, and BCL2L11 in the *EBF3*-overexpressing group were verified by Western blot analysis.

Because we observed that overexpression of *EBF3* induced apoptosis in leukemia cells, we examined the apoptosis-related genes by real-time PCR array that are implicated in *EBF3* overexpressing HL-60 cells, cells that harbor an empty vector, or a vector overexpressing *EBF3*. The real-time PCR array was composed of 370 key genes that have been associated with apoptosis (Figure 7A). Examination of the array data revealed that 62 genes were significantly upregulated and 31 genes were significantly downregulated in the *EBF3*-overexpressing group compared with the control group (Table 5 and Table 6, respectively). Genes that were most significantly upregulated and downregulated in response to *EBF3* overexpression are shown in Figure 7B and 7C, respectively. The up-regulated genes included cyclin-dependent kinase inhibitor 1A (p21, Cip1), DCC netrin 1 receptor, apoptosis-inducing factor, mitochondrion-associated 2, death-associated protein kinase 1 and 2, and caspase recruitment domain family, member 10. The down-regulated genes included zinc finger protein 443, baculoviral IAP repeat containing 8, and BCL2-like 11 (apoptosis facilitator). To validate the results of the real-time PCR array, we examined some of the dysregulated molecules at the protein level. The up-regulation of CDKN1A, DCC, and AIFM2 and the down-regulation of ZNF443, BIRC8, and BCL2L11 in *EBF3*-overexpressing group was verified by western-blot analysis (Figure 8).

**Table 5:** **Up-regulated genes in HL-60 cells treated with PLVX-** ***EBF3*** **compared with control group**

	**Gene**	**Description**	**Vector**	***EBF3***	**FC**	**P**
1	ANXA4	annexin A4	60.7992	65484.55	1077.063	0.000000
2	MTL5	metallothionein-like 5,	3327.433	78707.44	23.6541	0.004317
3	CDKN1A	cyclin-dependent kinase inhibitor 1A (p21, Cip1)	477.7772	9830.082	20.57462	0.005702
4	PRLR	prolactin receptor	166.5942	3355.185	20.13987	0.00595
5	DCC	DCC netrin 1 receptor	60.97703	1106.082	18.13932	0.00733
6	AIFM2	apoptosis-inducing factor, mitochondrion-associated, 2	5221.303	76648.86	14.68003	0.01117
7	BIRC7	baculoviral IAP repeat containing 7	10810.86	134973.2	12.48496	0.01541
8	APOE	apolipoprotein E	1234.913	15389.71	12.46218	0.015466
9	FAIM	Fas apoptotic inhibitory molecule	59.72215	723.2259	12.10985	0.016371
10	BCL6	B-cell CLL/lymphoma 6	300.2861	3465.696	11.54131	0.018009
11	EEF1A2	eukaryotic translation elongation factor 1 alpha 2	3430991	34629902	10.09326	0.023482
12	DAPK1	death-associated protein kinase 1	36.00672	341.4206	9.482138	0.026565
13	DAPK2	death-associated protein kinase 2	106.9056	926.7119	8.668504	0.031703
14	CUL-5	cullin 5	1607.044	10876.05	6.767734	0.021489
15	CARD10	caspase recruitment domain family, member 10	736432.7	4740088	6.436553	0.03677
16	HTATIP2	HIV-1 Tat interactive protein 2	21030.48	125463.2	5.965781	0.006577
17	HSPA1B	heat shock 70 kDa protein 1B	9411.403	51996.48	5.524838	0.046279
18	EIF2AK3	eukaryotic translation initiation factor 2-alpha kinase 3	1296.309	6741.561	5.200581	0.035667
19	PTH	parathyroid hormone	3.364038	14.63482	4.350374	0.020214
20	FAS	Fas cell surface death receptor				
	278.2416	1168.472	4.199489	0.028443		
21	PPP2CA	protein phosphatase 2, catalytic subunit	19486.59	77413.57	3.972658	0.012455
22	GFRAL	GDNF family receptor alpha like	2.948935	11.44733	3.881851	0.018696
23	CBX4	chromobox homolog 4	12247.45	47277.53	3.860193	0.010244
24	Bcl2-A1	BCL2-related protein A1	191.3664	733.6087	3.833528	0.012181
25	CASP7	caspase 7, apoptosis-related cysteine peptidase	3214.089	11901.59	3.702944	0.012218
26	AIFM3	apoptosis-inducing factor, mitochondrion-associated, 3	2320.458	8123.495	3.500815	0.019759
27	TNFAIP3	tumor necrosis factor, alpha-induced protein 3	396.2299	1312.111	3.311488	0.018799
28	PRODH	proline dehydrogenase (oxidase) 1	1017.064	3162.695	3.109634	0.022482
29	BIK	BCL2-interacting killer (apoptosis-inducing)	3304.449	10077.62	3.049712	0.020294
30	CFL1	cofilin 1	96630.71	284065.3	2.9397	0.024696
31	PIK3CA	phosphatidylinositol-4,5-bisphosphate 3-kinase	1192.848	3307.876	2.773091	0.027971
32	SERPINB2	serpin peptidase inhibitor, clade B	324.0773	898.5624	2.77268	0.022041
33	DAP3	death associated protein 3	59483.18	156832.7	2.636589	0.029577
34	MSH6	mutS homolog 6	10961.78	26493.5	2.416898	0.033269
35	XIAP	X-linked inhibitor of apoptosis	680.3787	1616.734	2.376227	0.033028
36	PRKRA	protein kinase, interferon-inducible double stranded RNA dependent activator	6339.679	15064.52	2.376227	0.035028
37	CD28	CD28 molecule	60.97703	140.9465	2.311469	0.039382
38	DEDD	death effector domain containing	10588.38	24135.46	2.27943	0.037863
39	CUL4A	cullin 4A	43243.45	98570.4	2.27943	0.037863
40	ERN1	endoplasmic reticulum to nucleus signaling 1	786.986	1766.819	2.245046	0.038268
41	PMAIP1	phorbol-12-myristate-13-acetate-induced protein 1	3492.862	7820.367	2.238957	0.038967
42	API5	apoptosis inhibitor 5	12078.84	26298.57	2.177243	0.040781
43	CHEK2	checkpoint kinase 2	11995.4	26047.67	2.171471	0.040504
44	CIDEC	cell death-inducing DFFA-like effector c	401.7611	872.4127	2.171471	0.040804
45	BAG3	BCL2-associated athanogene 3	6889.55	14849.39	2.155349	0.041371
46	CFLAR	CASP8 and FADD-like apoptosis regulator	1746.431	3747.656	2.145894	0.046262
47	TNFRSF19	tumor necrosis factor receptor superfamily, member 19	64.45383	136.2673	2.114186	0.042161
48	CASP5	caspase 5, apoptosis-related cysteine peptidase	439.6446	922.1559	2.097503	0.043498
49	MAPK8IP2	mitogen-activated protein kinase 8 interacting protein 2	200.8806	416.1298	2.071529	0.049992
50	BIRC3	baculoviral IAP repeat containing 3	129.8043	268.4701	2.068268	0.044074
51	BNIP3	BCL2/adenovirus E1B 19 kDa interacting protein 3	53609.24	110136	2.054421	0.044571
52	DPF2	D4, zinc and double PHD fingers family 2	40910.8	84044.57	2.054337	0.045739
53	HMGB-1	high mobility group box 1	299084.6	610176.4	2.040147	0.045059
54	CFDP1	craniofacial development protein 1	6517.912	13297.5	2.040147	0.045059
55	HDAC-1	histone deacetylase 1	75291.22	153605.1	2.040147	0.045559
56	CYCS	cytochrome c, somatic	6472.889	13109.45	2.025285	0.045684
57	NLRP1	NLR family, pyrin domain containing 1	32772.41	66367.88	2.025115	0.045743
58	BCLAF1	BCL2-associated transcription factor 1	10227.7	20698.39	2.023758	0.045215
59	DDX20	DEAD (Asp-Glu-Ala-Asp) box polypeptide 20	9090.816	18355.6	2.019137	0.045827
60	IKBIP	IKBKB interacting protein	3327.433	6694.993	2.012059	0.040311
61	EDAR	ectodysplasin A receptor	1090.062	2191.093	2.010063	0.046015
62	MAPK8	mitogen-activated protein kinase 8	8842.227	17718	2.003793	0.043235

**Table 6:** **Down-regulated genes in HL-60 cells treated with PLVX-** ***EBF3*** **compared with control group**

	**Gene**	**Description**	**Vector**	***EBF3***	**FC**	**P**
1	ZNF443	zinc finger protein 443	199.493	3.80756	0.019086	0.000602
2	BIRC8	baculoviral IAP repeat containing 8	433.5919	29.62754	0.06833	0.007686
3	BCL2L11	BCL2-like 11 (apoptosis facilitator)	8365.257	663.1	0.079268	0.01033
4	CARD17	caspase recruitment domain family, member 17	28.84389	2.494701	0.08649	0.012285
5	NLRP2	NLR family, pyrin domain containing 2	16.79773	1.462936	0.087091	0.012456
6	NTF3	neurotrophin 3	910.2974	122.6894	0.134779	0.029574
7	INHA	inhibin, alpha	2435.824	404.1836	0.165933	0.044486
8	IL10	interleukin 10	115.3756	24.23243	0.210031	0.007331
9	FADD	Fas (TNFRSF6)-associated via death domain	34164.11	8072.876	0.236297	0.008898
10	CD5	CD5 molecule	233.9723	60.92097	0.260377	0.010608
11	SNCA	synuclein, alpha	38703.97	10360.94	0.267697	0.011798
12	SST	somatostatin	54.95558	15.65842	0.284929	0.015721
13	SERPINB9	serpin peptidase inhibitor, clade B	4153.737	1191.751	0.286911	0.017364
14	PIDD	p53-induced death domain protein	25712.71	7480.23	0.290916	0.0130711
15	TPD52L1	tumor protein D52-like 1	621.7514	187.2558	0.301175	0.013438
16	RARG	retinoic acid receptor, gamma	2628.81	808.3672	0.307503	0.01493
17	ALOX15B	arachidonate 15-lipoxygenase, type B	8365.257	2700.231	0.322791	0.015529
18	PAK7	p21 protein (Cdc42/Rac)-activated kinase 7	657.2025	219.6202	0.334174	0.016946
19	LCK	LCK proto-oncogene, Src family tyrosine kinase	17082.06	5788.071	0.338839	0.017284
20	ADRA1D	adrenoceptor alpha 1D	247.313	91.70109	0.37079	0.024013
21	PCBP4	poly(rC) binding protein 4	9675.993	3587.758	0.37079	0.024013
22	TNFRSF18	tumor necrosis factor receptor superfamily, member 18	3420.98	1340.789	0.391931	0.0225247
23	GRM4	glutamate receptor, metabotropic 4	576.1074	233.7572	0.405753	0.023978
24	CRYAA	crystallin, alpha A	1184.608	490.7576	0.414278	0.024834
25	FASLG	Fas ligand (TNF superfamily, member 6)	233.9723	99.65477	0.425925	0.026701
26	ALOX12	arachidonate 12-lipoxygenase	153.2979	66.31	0.432556	0.026786
27	PEA15	phosphoprotein enriched in astrocytes 15	3083.16	1350.115	0.4379	0.027532
28	S100B	S100 calcium binding protein B	4211.721	1896.163	0.450211	0.028891
29	NLRP3	NLR family, pyrin domain containing 3	16614.95	7797.884	0.469329	0.037943
30	TNFRSF1B	tumor necrosis factor receptor superfamily, member 1B	3984.531	1936.005	0.48588	0.036438
31	TNFRSF8	tumor necrosis factor receptor superfamily, member 8	7487.114	3740.115	0.49954	0.034869

### Ingenuity pathway analysis tool displays a pathway regulated by *EBF3* overexpression in HL-60 cells

**Figure 9:**
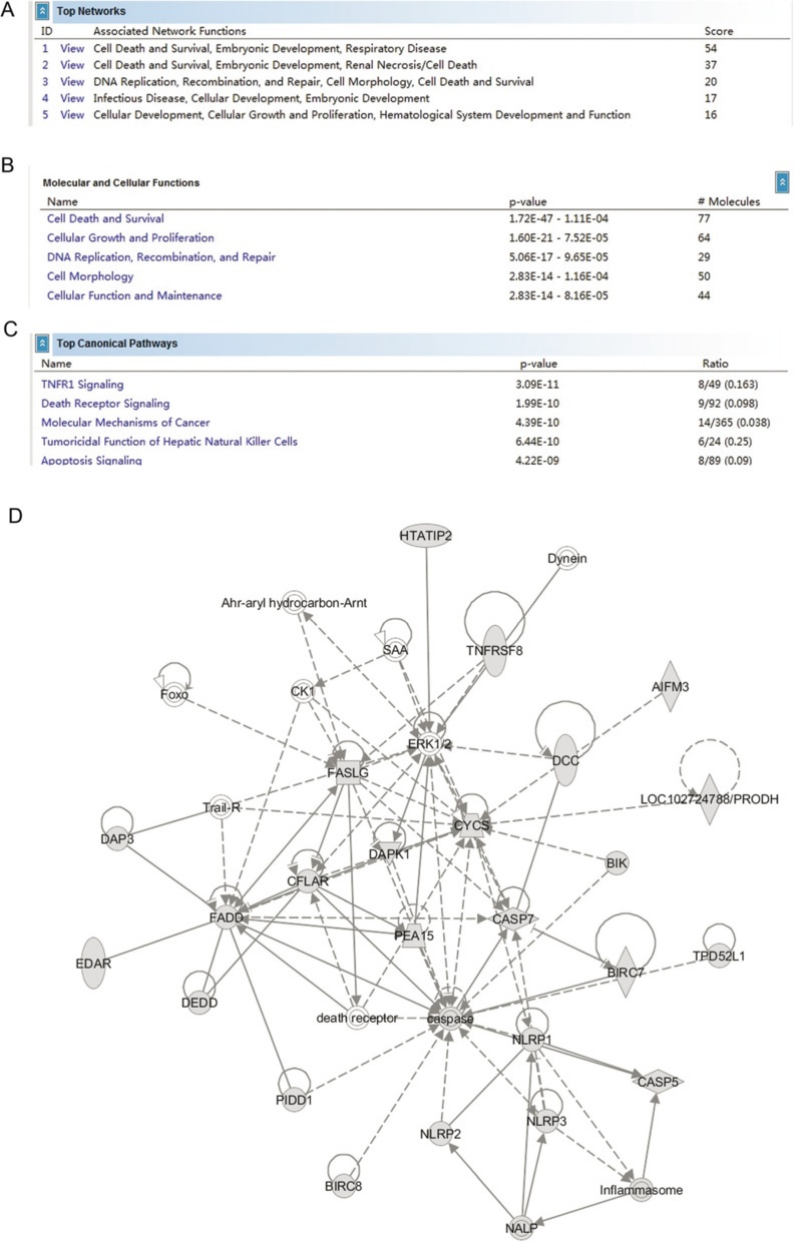
**IPA summary of the pathways regulated by** ***EBF3*** **overexpression in HL-60 cells.** Datasets representing 370 key genes involved in apoptosis with altered expression profiles that were obtained from real-time PCR arrays were imported into the IPA Tool and the following data is illustrated: **(A)** A list of the top five networks with their respective scores obtained from IPA. **(B)** A list of the top five molecular and cellular functions with their respective scores obtained from IPA. **(C)** A list of the top five canonical pathways with their respective scores obtained from IPA. **(D)** The network representation of the most highly rated network is shown. Genes that are shaded were determined to be statistically significant. A solid line represents a direct interaction between the two gene products and a dotted line means an indirect interaction.

To investigate the possible biological interactions of differentially regulated genes, datasets representing genes with altered expression profiles derived from our real-time PCR array analysis were imported into the Ingenuity Pathway Analysis (IPA) Tool. The list of differentially expressed genes analyzed by IPA revealed significant networks. Figure 9A depicts the list of the top 5 networks identified by IPA. Of these networks, cell death was the highest rated network with 77 focus molecules and a significance score of 54 (Figure 9D). The score is the probability that a collection of genes equal to or greater than the number in a network could be achieved by chance alone. A score of three indicates a 1/1000 chance that the focus genes are in a network not due to random chance. Figure 9D indicated firstly that ERK1/2, DAPK1, and caspase might be related to the *EBF3* pathway. The IPA analysis also groups the differentially expressed genes into biological mechanisms that are related to cell death and survival, cellular growth and proliferation, DNA replication, cell morphology and cellular function, and maintenance (Figure 9B). The top five most significant pathways were presented in Figure 9C. These results may provide new clues for the molecular mechanisms of apoptosis induced by *EBF3* overexpression.

Pediatric AML is a heterogeneous disease, which currently can be cured in approximately 70% of children. Five-year survival of pediatric AML varies from 15% – 70%, relapse rate varies from 33% – 78%, and its incidence is expected to increase. Hypermethylation of *EBF3* has been reported in patients with rheumatoid arthritis [33], gastric carcinoma [26], head and neck squamous cell carcinoma [34], and neoplasms of the pancreas [35]. In this study, we found that the *EBF3* promoter was hypermethylated in pediatric AML. Our results showed that the *EBF3* promoter was hypermethylated in 10/12 leukemia cell lines. PCR analysis showed that *EBF3* expression was significantly upregulated in leukemia cells following 5-Aza treatment compared with control cells treated with DMSO. In addition, western blot analysis showed that expression of *EBF3* in NBM samples (n = 8) was significantly higher than leukemia cell lines (n = 9); these results are consistent with the MSP assay in leukemia cell lines. Aberrant methylation of *EBF3* was observed in 39.0% (16/41) of pediatric AML samples compared with 6.7% (2/30) of NBM control samples. Consistent with the MSP results, the BGS results confirmed that the CpG islands in the *EBF3* promoter were methylated in the AML samples (67.0% - 77.0%), whereas they were unmethylated in the NBM samples (41.0% - 50.0%). Taken together, these results imply that methylation may be involved in the downregulation of *EBF3* in pediatric AML.

Although the *EBF3* locus on chromosome 10q26.3 is epigenetically silenced or deleted in several types of cancers, the prognostic value of *EBF3* has only been reported in gastric carcinoma. Promoter methylation of *EBF3* was found to be significantly correlated with lymphatic invasion (p = 0.013) and poor survival (p = 0.038) in gastric carcinoma. These results suggest that the *EBF3* tumor suppressor is epigenetically silenced and that it serves as an independent prognostic marker in gastric carcinoma [26]. Our results showed no significant differences in clinical features, such as sex, age, initial hemoglobin level, white blood cell counts, platelet counts and chromosomal abnormalities, between patients with methylated *EBF3* and those with unmethylated *EBF3* by examination of the clinicopathologic characteristics. Samples with *EBF3* promoter methylation revealed similar survival outcomes through Kaplan-Meier survival analysis. Multivariate analysis revealed that *EBF3* promoter methylation is not an independent prognostic factor in pediatric AML. This study represents the first report showing that the methylation of *EBF3* is not an independent predictor of poor survival in AML.

A previous study in brain tumors revealed that expression of *EBF3* resulted in cell cycle arrest and apoptosis. The expression of cyclin-dependent kinase inhibitors was profoundly affected with early activation of p21 (cip1/waf1) and persistent activation of both p27 (kip1) and p57 (kip2), whereas genes involved in cell survival and proliferation were suppressed [25]. Until now, the molecular function of *EBF3* in AML cells is still unknown. Our results firstly indicated that *EBF3* overexpression significantly inhibits cell proliferation. The proportion of apoptotic cells in the *EBF3*-overexpressing cells was significantly greater than that of the vector control group in both HL-60 and MV4-11 cells. The apoptotic effect of *EBF3* in HL-60 and MV4-11 cells was confirmed; we investigated the expression levels of cleaved PARP, a marker of apoptosis by Western blot. The results were consistent with the Annexin V data, confirming that *EBF3* induced apoptosis in AML leukemia cells.

Real-time PCR array analysis is an effective technique for quantifying the expression of a focused panel of genes [36,37]. Therefore, to explore the underlying mechanisms of the *EBF3* antitumor activity, we carried out a real-time PCR array assay on 370 apoptosis-related genes to identify genes that were dysregulated in AML following *EBF3* overexpression. The findings showed that 62 genes were significantly upregulated and 31 genes were significantly downregulated in the *EBF3*-overexpressing group compared with the control group. Some genes, such as *CDKN1A* and *PARP1* cleavage, have been reported with EBF3, while *DCC*, *AIFM2*, and *DAPK1* have never been reported with *EBF3*. The *DCC* gene encodes a netrin 1 receptor, partially localizes to lipid rafts, and induces apoptosis in the absence of ligand. DCC functions as a tumor suppressor and is frequently mutated or downregulated in colorectal [38] and ovarian cancers [39]. AIFM2 encodes a flavoprotein oxidoreductase that binds single stranded DNA and is thought to contribute to apoptosis in the presence of bacterial and viral DNA. Overexpression of AIFM2 induced cell death with characteristic apoptotic morphology, and the apoptosis was independent of caspase activation and p53 and was not inhibited by Bcl-2. These findings suggest that AIFM2 induces a novel caspase-independent apoptotic pathway [40,41]. DAPK1 is a positive mediator of interferon-gamma induced programmed cell death. Overexpression of DAPK1 in various cell lines results in cell death [42]. Our research firstly implied that DCC, AIFM2, and DAPK1 might be novel targets of *EBF3*.

The basis of the IPA program consists of the Ingenuity Pathway Knowledge Base (IPKB), which is derived from known functions and interactions of genes published in the literature. The IPA Tool allows the identification of biological networks, global functions, and functional pathways of a particular dataset. This work shows that cell death was the highest rated network with 77 focus molecules and a significance score of 54. In addition, this study was the first to indicate that ERK1/2, DAPK1, and caspase may be related in the *EBF3* pathway. However, the mechanism and the role of these genes in *EBF3*-induced apoptosis in AML remain to be elucidated further.

## Conclusions

In this study, we identified epigenetic inactivation of *EBF3* in both AML cell lines and pediatric AML samples for the first time. The expression of *EBF3* was significantly lower in pediatric AML compared with control samples. In addition, our findings showed for the first time that transcriptional overexpression of *EBF3* could inhibit proliferation and induce apoptosis in AML cells. We identified 93 dysregulated apoptosis-related genes in *EBF3*-overexpressing cells, including *DCC*, *AIFM2*, and *DAPK1*. Most of these genes have never been related to *EBF3* overexpression. These results may provide new insights into the molecular mechanism of *EBF3*-induced apoptosis; however, further research will be required to determine the underlying details. Taken together, our findings suggest that *EBF3* may act as a putative tumor suppressor gene in pediatric AML.

## Abbreviations

ALL: Acute lymphoblastic leukemia
AML: Acute myeloid leukemia
MSP: Methylation specific PCR
BGS: Bisulfite genomic sequencing
NBM: Normal bone marrow
ITP: Idiopathic thrombocytopenic purpura
